# Genus trace reveals the topological complexity and domain structure of biomolecules

**DOI:** 10.1038/s41598-018-35557-3

**Published:** 2018-12-03

**Authors:** Sebastian Zając, Cody Geary, Ebbe Sloth Andersen, Pawel Dabrowski-Tumanski, Joanna I. Sulkowska, Piotr Sułkowski

**Affiliations:** 1 Faculty of Mathematics and Natural Studies, Cardinal Stefan Wyszynski University, Dewajtis 5, 01-815 Warsaw, Poland; 2 Interdisciplinary Nanoscience Center, Aarhus University, DK-8000 Aarhus C, Denmark; 3 Department of Bioengineering, California Institute of Technology, Pasadena, CA 91125 USA; 4 Department of Molecular Biology and Genetics, Aarhus University, DK-8000 Aarhus C, Denmark; 5 Faculty of Chemistry, University of Warsaw, Pasteura 1, 02-093 Warsaw, Poland; 6 Centre of New Technologies, University of Warsaw, Banacha 2c, Warsaw, 02-097 Poland; 7 Faculty of Physics, University of Warsaw, Pasteura 5, 02-093 Warsaw, Poland; 8 Walter Burke Institute for Theoretical Physics, California Institute of Technology, Pasadena, CA 91125 USA

## Abstract

The structure of bonds in biomolecules, such as base pairs in RNA chains or native interactions in proteins, can be presented in the form of a chord diagram. A given biomolecule is then characterized by the genus of an auxiliary two-dimensional surface associated to such a diagram. In this work we introduce the notion of the genus trace, which describes dependence of genus on the choice of a subchain of a given backbone chain. We find that the genus trace encodes interesting physical and biological information about a given biomolecule and its three dimensional structural complexity; in particular it gives a way to quantify how much more complicated a biomolecule is than its nested secondary structure alone would indicate. We illustrate this statement in many examples, involving both RNA and protein chains. First, we conduct a survey of all published RNA structures with better than 3 Å resolution in the PDB database, and find that the genus of natural structural RNAs has roughly linear dependence on their length. Then, we show that the genus trace captures properties of various types of base pairs in RNA, and enables the identification of the domain structure of a ribosome. Furthermore, we find that not only does the genus trace detect a domain structure, but it also predicts a cooperative folding pattern in multi-domain proteins. The genus trace turns out to be a useful and versatile tool, with many potential applications.

## Introduction

The structure of biopolymers is primarily defined by complex three-dimensional networks of bonds or contacts of nucleobase or peptide units. In RNA and DNA, these interactions take the form of hydrogen bonds that link nucleotides together at their edges, and base-stacking, that make the nucleotide units pack into a tight helix. The hydrogen bonds define whether the RNA folds into a single-strand, a hairpin, or more complex branched structures as is often the case. In proteins, contacts formed between peptide units affect the whole process of protein folding and lead to the resulting three-dimensional structure of proteins.

Here, we introduce the *genus trace*, a novel way to rank and quantify the complexity of biomolecules based on the entanglement of bonds or contacts as they form along the backbone chain. The genus trace measures the increase in complexity of the network of bonds or contacts connecting the residues along the biomolecule chain. For example, in the context of RNA secondary structure interactions such as hairpins, loops, bulges, and junctions do not alone contribute to the genus trace, but it is the contacts between these elements in three-dimensional space that increase the genus. Therefore, the genus trace represents how interconnected and densly packed the structure is in three-dimensions. Moreover, the genus trace provides an interesting perspective on RNA and protein folding: it enables one to imagine sitting at the “synthesis end” and watching how the molecule folds up during synthesis – or, alternatively, taking the roller coaster trip through a crystal structure to see how its complexity builds up.

In this work we seek to demonstrate how the genus trace can be used to rank the complexity of different biological structures, and to identify regions of high structural complexity such as different structural domains. Our analysis is not restricted to biomolecules, and can be applied to interactions within any polymer-like system of chains. Nonetheless, when discussing specific applications, we focus on biomolecules, which provide a huge set of examples and for which our methods can be particularly useful.

## Genus and Biomolecules

Before introducing the *genus trace*, we recall what the *genus* is and how it can be used in the analysis of biopolymers. Note that the genus of RNA structures was considered before, e.g. in^1–8^, or for proteins in^9^. However in those works the genus was computed only for the entire chain length, and taking into account only canonical Watson-Crick base pairs in the RNA case. Here we show that much more detailed information is revealed once genus is computed for various types of bonds in a given structure, e.g. also for non-canonical base pairs, including those involved in helix backbone packing interactions in RNA. Moreover, the genus trace that we introduce in what follows captures much more information than solely the genus of the whole chain.

### What is genus and how to compute it?

**Figure 1:**
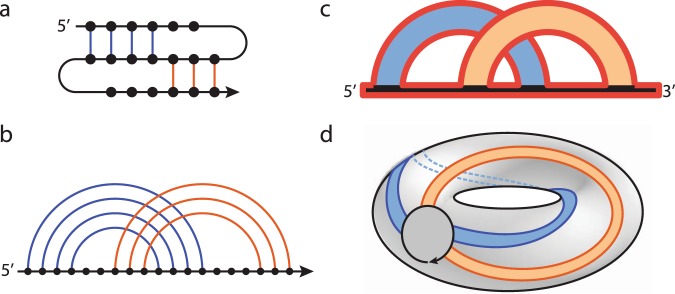
How to compute the genus. (**a**) A chain with several bonds (in blue and orange) connecting various pairs of residues (black dots). (**b**) Chord diagram representing the same structure. (**c**) Parallel chords replaced by a single chord, and then – together with the backbone – replaced by ribbons, whose single boundary is shown in red. (**d**) After shrinking the backbone to a small circle, the ribbon diagram can be smoothly drawn on a surface of a torus, whose genus is *g* = 1.

Consider a polymer-like chain consisting of a number of residues, with bonds connecting various pairs of these residues, as in the example in Fig. 1(a). The structure of such a chain can be presented in the form of a chord diagram. A chord diagram consists of *b* horizontal intervals (called backbones) that represent one or more polymer-like chains, and *n* arcs (chords) representing bonds, which connect pairs of residues, and are drawn as half-circles in the upper-half plane. In this work we consider configurations with only one backbone, $b=1$. A chord diagram corresponding to the structure in Fig. 1(a) is shown in Fig. 1(b). Such diagrams are commonly used to present the structure of RNA chains^3,4^. A stack of parallel chords contributes in the same way as a single chord to the genus, so each set of parallel chords can be replaced by one chord, as in Fig. 1(c). Furthermore, to compute the genus it is of advantage to replace all backbones and chords by ribbons of finite width, also as in Fig. 1(c). In this way we obtain a two-dimensional surface with *r* boundaries, which – after shrinking a backbone to a small circle – can be drawn in a smooth way on an auxiliary surface of genus *g* (i.e. having *g* “holes”), as in Fig. 1(d). The genus of a chord diagram is defined as the genus of this auxiliary surface. This genus can be determined from the Euler formula

1 $$b-n=2-2g-r.$$

For example, in Fig. 1(c) there is $b=1$ backbone, $n=2$ chords, and $r=1$ boundary (drawn in red). Therefore it follows from the Euler formula that the genus $g=1$, so that the auxiliary surface is a torus, see Fig. 1(d).

Note that if no chords intersect in a given chord diagram then $g=0$; in this case the chord diagram is called planar. In particular, a large complicated RNA with a secondary structure having all nested basepairs has genus $g=0$, so it is quite simple from the point of view of this paper. Furthermore, for a fixed number of chords and backbones the genus cannot exceed some maximal value. We also recall that chord diagrams are used by mathematicians to characterize moduli spaces of Riemann surfaces, while physicists reinterpret them as a particular class of Feynman diagrams arising in certain quantum field theories or matrix models^4,7^. Certain properties of chord diagrams have been also discussed in^10^.

### Types of bonds and bifurcations

**Figure 2:**
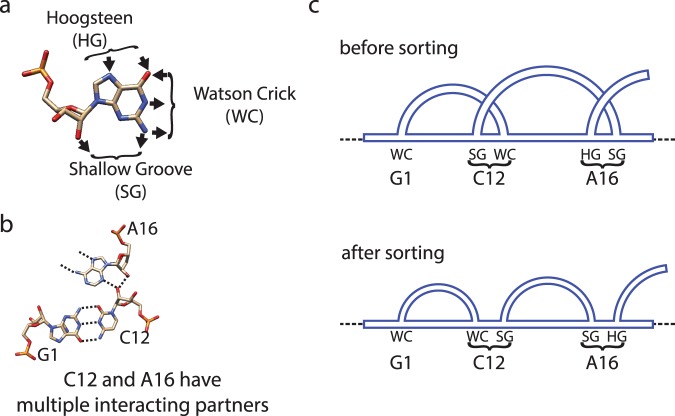
Resolving bifurcations. (**a**) Each nucleotide has three edges: Hoogsteen (denoted HG or H), Watson-Crick (WC or W), and Shallow Groove or Sugar (SG or S). (**b**) An example of a bifurcation in RNA structure. (**c**) Resolving a bifurcation: overlapping chord endpoints are replaced by separate residues, which are then sorted so that no additional crossings are introduced.

To determine the genus, for example using the formula (1), one simply considers all bonds in a given chain. However in various contexts, in particular for biomolecules, one can distinguish between various types of bonds. In this work we propose to consider such a distinction; as we will see, this provides some new information about those different types of bonds. For RNA, an important classification of base pairs have been introduced by Leontis and Westhof^11,12^. They noticed that RNA bases can be regarded as triangles with three different edges, referred to as: Hoogsteen edge (denoted HG or H), Watson-Crick edge (denoted WC or W), and Sugar or Shallow Groove edge (denoted S or SG), see Fig. 2(a). Base pairs are formed by any of these three edges of a nucleotide with an edge of another nucleotide. Depending on the orientation, a given base pair may have a configuration *trans* (t) or *cis* (c). A given base pair is denoted by specifying its orientation and edges it involves; for example, cWW denotes base pairs formed by two Watson-Crick edges in the *cis* configuration. Altogether there are 12 types of base pairs, which we list here in the order corresponding to the frequency of their occurrence^13^, and which will be important in what follows:

2 $$\begin{array}{l}{\rm{cWW}},{\rm{tHS}},{\rm{tWH}},{\rm{tSS}},{\rm{cWS}},{\rm{tWS}},\\ {\rm{cHS}},{\rm{tWW}},{\rm{cWH}},{\rm{tHH}},{\rm{cSS}},{\rm{cHH}}.\end{array}$$

For proteins one can also consider various types of contacts, e.g. between various secondary structures: between two helices, or two *β*-strands, or between a helix and a *β*-strand.

In the computation of the genus for biomolecules we have to take into account an important subtlety, that we refer to as bifurcations – namely, a given residue may form bonds with more than one other residue. For example, in RNA a given nucleotide may form base pairs with more than one other nucleotide, as in Fig. 2(b), and in proteins a given amino acid may be in contact with more than one other amino acid. In the language of chord diagrams this means that more than one chord is attached in the same place of the backbone, which is not an allowed configuration, and in such case the genus cannot be computed. To deal with this subtlety we split each residue into as many residues as the number of bonds it forms, and sort endpoints of chords in such a way, that no intersections are introduced in the corresponding chord diagram by chords ending in those residues. This ensures that the genus can be computed and it is not artificially increased (by “artificially” introduced crossings), and it has a well defined minimal possible value. An example of such sorting, in the case of the bifurcation shown in Fig. 2(b), is shown in Fig. 2(c).

### Genus classification of RNA structures

Once we know how the genus is defined, it is of interest to compute it for all known RNA structures. Such a computation, however taking into account only canonical (cWW) base pairs, was first conducted in^3^. Here we show that taking into account other types of base pairs reveals much more interesting information. Moreover, since the work of  ^3^ many more RNA structures have been identified, so it is also of advantage to compute genus for all of them.

**Figure 3:**
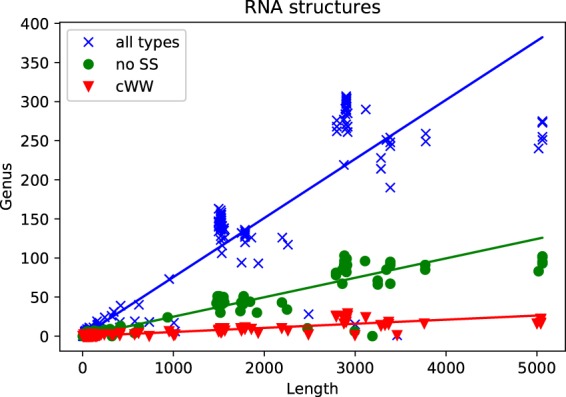
Genus computed for all known RNA structures. Each point represents one structure, and its coordinates denote respectively its length and genus. Red triangles correspond to genus computed taking into account only canonical (cWW) base pairs. Green dots represent genus computed for all base pairs apart from sugar-sugar (cSS and tSS) interactions. Blue ×’s correspond to genus computed for all base pairs.

We computed genus for RNA structures with better than 3.0 Å resolution in the PDB database (also deposited in the BGSU RNA Site database^14^). Out of the total of 1240 structures, 565 did not contain errors and were of appropriate form for the genus computation (from the first backbone in the structure). In genus computations we considered three classes of base pairs. First, we considered only canonical (cWW) base pairs. Second, we considered all possible base pairs excluding the sugar-sugar (cSS and tSS) base pairs, as SS interaction are largely responsible for helix packing interactions and therefore necessarily introduce crossing chords that increase the genus. Third, we took into account all possible base pairs listed in Eq. (2). Results for these three classes are shown in Fig. 3, respectively by red triangles (cWW interactions), green dots (all but sugar-sugar interactions), and blue crosses (all base pairs). Each triangle, a dot, or a cross corresponds to one RNA chain, and its coordinates correspond respectively to the length (the number of nucleotides) of this chain and the genus (of the corresponding auxiliary surface).

From the data in Fig. 3 several conclusions can be drawn. First, sugar-sugar interactions indeed contribute significantly to the genus, as expected. Second, for each three classes of base pairs, the dependence of the genus *g* on the length of the chain *d* (the number of nucleotides) is linear to a good approximation – as shown in Fig. 3 – and takes the form:

3 $$\begin{array}{ll}{\rm{Only}}\,\mathrm{cWW}: & g=0.005d,\\ {\rm{All}}\,{\rm{but}}\,\mathrm{SS}: & g=0.040d,\\ {\rm{All}}\,{\rm{base}}\,\mathrm{pairs}: & g=0.076d.\end{array}$$

The slopes 0.040 and 0.076 are 8 and 15 times higher than the slope with cWW only, so the genus indeed depends significantly on non-canonical base pairs. Moreover, note that the result for canonical base pairs (red triangles) is of the same order as the slope $\simeq$0.003 found in^3^. However, our computation involves many more RNA structures known as of 2018, and the linear character of the resulting plot in Fig. 3 is much more evident than of the plot in^3^.

Moreover, in Fig. 3 it is clearly seen that RNA structures are divided into 3 main groups: those of length shorter than 1000 nucleotides (450 structures), those of length between 1000–2500 (72 structures), and those of length above 2500 nucleotides (43 structures). The second and the third groups correspond respectively to small and large subunits of ribosome structures, whose genus is very large. It is in the range $50\, < \,g < \,200$ for the following 71 structures:

1FJG, 1IBL, 1N32, 1N33, 1XNQ, 1XNR, 2F4V, 2UUA, 2UXB, 2UXC, 2UXD, 2VQE, 3J7Y, 3J9M, 3J9W, 3JAM, 3JBU, 3JBV, 3JCS, 3JCT, 3T1Y, 4B3T, 4BTS, 4DR6, 4DR7, 4GKJ, 4GKK, 4JV5, 4JYA, 4K0K, 4KHP, 4TUE, 4U26, 4V19, 4V4Q, 4V50, 4V5G, 4V5K, 4V6E, 4V7M, 4V83, 4V84, 4V85, 4V8N, 4V8U, 4V92, 4V9I, 4V9L, 4V9R, 4W29, 4WSM, 4XEJ, 5A2Q, 5AJ3, 5AN9, 5E7K, 5E81, 5EL4, 5IB8, 5IBB, 5IT7, 5IT9, 5J7L, 5JU8, 5JUP, 5MC6, 5O5J, 5T2A, 5T5H, 5T7V, 5TCU

and in the range *g* ≥ 200 for 40 structures:

1NJP, 1QVF, 1S72, 1VQ6, 1VQL, 1VQM, 1VQN, 1VQO, 3J6B, 3J79, 3J7P, 3J7Q, 3J7R, 4IOA, 4U4U, 4UG0, 4V88, 4V8C, 4V8E, 4V8P, 4V8Q, 4V91, 4V9F, 4V9Q, 4WFA, 5AJ0, 5DM6, 5FDU, 5HL7, 5L3P, 5LZD, 5MGP, 5MMI, 5MMM, 5MRC, 5ND8, 5O61, 5T2C, 5UMD, 5X8P

Most of the remaining structures with at most 1000 chords have much lower genus (computed for all base pairs): for 229 structures the genus is simply $g=0$, for 225 structures it is lower than 50.

Note that when all base pairs (with or without sugar-sugar interactions) are taken into account, the value of genus grows significantly and for small or large subunits of the ribosome it is of order of several hundreds. These values are much larger than for genus computed only for canonical base pairs, which is of the order 20–30, even for long large ribosome units. As we will see in what follows, the properties of the genus trace are most interesting when the genus of the whole chain takes large values. For this reason long ribosomal subunits with all non-canonical base pairs will be of our particular interest in the following analysis.

We also computed the genus for designed artificial RNA structures, such as the “Peano curve” or the “smiley face”^15,16^. However, even though these structures are quite long (and considering all base pairs), their genus does not exceed 20. This is so, because these artificial structures do not take advantage of as many tertiary motifs to achieve a 3D compaction compared to natural RNA structures. Since the artificial structures consist of only cWW interactions, they end up having fairly low genus compared to natural folded RNAs of similar length.

## Genus Trace for Biomolecules

As we explained in the previous section, the genus is simply a number associated to a given chain. Now we introduce a function that we call the genus trace. We discuss its properties and compute it for all RNA and several characteristic protein chains. We show that it captures interesting structural, physical and biological information of those chains; in particular we find the genus trace detects domain structure of biomolecules.

### What is the genus trace and how to compute it?

We define the genus trace as a function *g*(*i*) that encodes the genus of all subchains contained from the first to the *i*’th residue in a given chain. The genus trace characterizes a complexity of bonds and the whole chain much more accurately than simply one number representing the genus of the whole chain.

**Figure 4:**
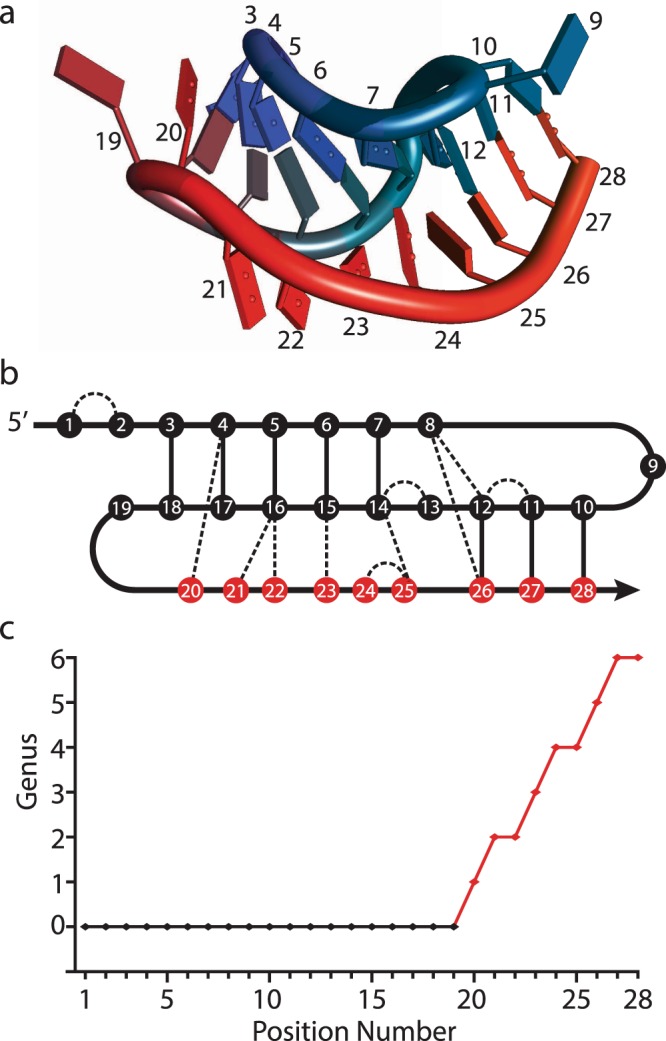
The genus trace. (**a**) Cartoon representation of RNA structure with PDB code 437D. (**b**) Schematic diagram of base pairs in this structure. (**c**) A plot of the genus trace.

We illustrate how to compute the genus trace in Fig. 4, in the example of RNA structure with PDB code 437D, shown in panel a). Its simplified diagram with all interactions is shown in panel b). Chords corresponding to all bonds in the range between the 1st and 20th nucleotide do not intersect, so the genus *g*(*i*) is zero for $i=1,\ldots ,19$. The base pair between the 4’th and 20’th nucleotides is the first one that contributes to non-zero genus, so that $g(20)=1$. The base pair between 16’th and 21’st nucleotide also increases the genus by one, hence $g(21)=2$. The resulting plot of the genus trace *g*(*i*) is shown in panel c). Note that in order to determine *g*(*i*) we had to resolve several bifurcations in this structure, as we explained earlier.

The genus trace has various interesting properties. If no chords (representing bonds) intersect in some range of *i*, then *g*(*i*) is constant in this region. On the other hand, if many chords in some localized region intersect – for example in case of complicated pseudoknots in RNA – then *g*(*i*) tends to grow rapidly.

### Base pairs in RNA and the ribosome structure

Having introduced the genus trace, we now use it to analyze real RNA structures and present what information it captures. In this analysis we distinguish between various types of base pairs in a given chain, which reveals additional information. The genus may change only by integer values, so when the genus of the whole chain attains large values then there is much room to observe variability of the genus trace, and so the genus trace analysis is then particularly interesting. As follows from our earlier analysis and data in Fig. 3, it is most interesting to analyze ribosomes, whose total genus is of order of several hundreds. There are several ribosomal structures available in the Protein Data Bank, which all lead to similar results; in this section we focus on one representative structure of large ribosomal subunit from *Haloarcula marismortui*, of PDB ID 1s72. The total genus for this structure (for the whole chain and taking into account all base pairs) equals 298.

**Figure 5:**
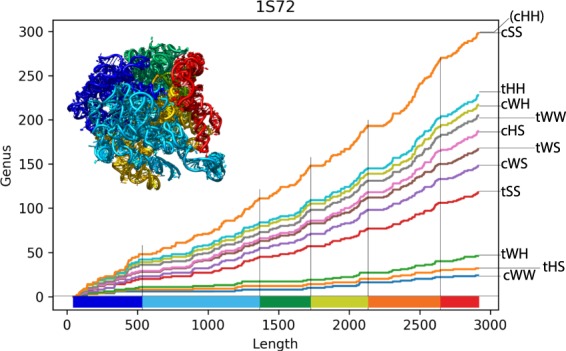
Specific features of various types of base pairs captured by genus plots for structure of large ribosomal subunit of the *Haloarcula marismortui* (PDB ID 1s72). Each new plot (from the bottom to the top) is obtained upon adding an additional type of base pairs to the analysis, according to the ordering in (2). Flat regions which are formed detect a domain structure of this ribosome; various colors on the horizontal axis correspond to domains, which are shown in the same color in the cartoon representation of the ribosome. The largest spaces between plots arise after including tSS and cSS detect helix packing and A-minor interactions, which indeed contribute most to the complexity of bonds. The most narrow spaces between plots correspond to rare cHH and tHH interactions.

We compute a contribution of various base pairs to the genus of 1s72 as follows. First, we fix the ordering of all possible base pairs as listed in Eq. (2), according to the probability of their formation^13^. Then, in the first step we choose the first type of a base pair in this list, i.e. cWW, and determine the genus trace *g*(*i*), which is shown as the bottom (blue) plot in Fig. 5. In the second step we determine *g*(*i*) taking into account the first two types of base pairs in the above list, i.e. cWW and tHS, which results in the second to bottom (orange) plot in Fig. 5. In the third step we determine *g*(*i*) taking into account cWW, tHS and tWH base pairs, etc. Ultimately we obtain a series of plots shown in Fig. 5. The orange top plot represents *g*(*i*) computed taking into account all base pairs in this ribosome chain.

The genus trace plots shown in Fig. 5 capture the following information. First, note that those plots – in particular the top one, determined from all base pairs – have several flat regions. It turns out that locations of these flat regions precisely correlate with division of the whole ribosome into distinct domains, as denoted by vertical black lines. This shows that the genus trace contains information about domain structures of large biomolecules. Indeed, a domain is just a subgrouping of interactions that are tightly clustered, and so there should naturally be a lower density of crossing interactions between clusters. Nonetheless, to some extent this is also dependent on how the domains interact. The pauses in-between the domains indicate that the domains are organized in a structure like beads on string, and the genus trace characterizes this structure quantitatively.

Another important feature of the genus trace that we discover is a difference between heights of plots for various types of base pairs. In particular the largest space appears between the third and the fourth (red) plot from the bottom, i.e. once tSS interactions are taken into account, which are responsible for helix packing. Moreover, the second largest spacing between plots arises once cSS base pairs, responsible for A-minor interactions, are included in the genus counting, as represented by the (almost invisible) second to top (grey) plot. These two types of interactions introduce complicated structures (with non-canonical crossing interactions) that significantly increase the genus trace, as indeed seen in Fig. 5. Furthermore, the very top (orange) plot overlaps with the second to the top plot, which is a manifestation of the fact that cHH base pairs are rarely observed in the PDB. Moreover, the third plot from the top (in blue) corresponds to adding tHH interactions which are very rare, which is the reason why the space below it is also very narrow. Furthermore, even though cWW are the most numerous base pairs, they contribute little to the genus (i.e. only cWW pseudoknots contribute to the genus), and so the cWW curve (in blue) is low.

We also note that it is not the ordering of base pairs chosen in (2) which is most crucial from the viewpoint of the genus trace, but the (relative) area between consecutive plots; such area characterizes how a given base pair type contributes to complexity of given structure. For 1s72 and a few other structures, the relative area (in percentage) between consecutive plots, characterizing a given base pair type, is given in Table 1.

**Table 1:** Relative area (in percentage) between consecutive genus trace plots, characterizing a given base pair type, for a few RNA structures.

Structure	cWW	tHS	tWH	tSS	cWS	tWS	cHS	tWW	cWH	tHH	cSS	cHH
1s72	8.21	2.94	3.96	24.29	9.74	7.73	3.66	7.26	5.04	3.70	23.48	0.00
1vqm	8.20	3.58	4.25	23.74	9.38	7.63	3.11	7.16	4.41	3.72	24.82	0.00
3j79	6.13	4.03	4.45	20.46	11.55	8.74	4.55	6.20	2.72	4.98	26.19	0.00
4ioa	7.44	5.21	5.25	26.96	6.35	8.68	3.66	5.87	4.08	3.85	22.64	0.00
5fdu	7.16	3.22	4.75	25.91	6.97	7.63	4.76	4.89	3.44	3.93	27.08	0.26

We see that the contribution of a given base pair type is similar for all these structures. The quantitative results also confirm that tSS and cSS base pairs contribute most to complexity of these RNA structures.

We have conducted such genus trace analysis for various other long RNA chains, which led to analogous results. We have found that the genus trace detects domain structure of those chains, and confirmed that sugar-sugar interactions significantly contribute to their complexity. In conclusion, the genus trace captures new information about the three-dimensional interconnections of base pairs that are not represented by bond type or geometry alone, and enables their quantitative description.

### Genus trace of proteins

Another broad area where the genus trace provides a new viewpoint and analytical tools is the realm of proteins. In this case we identify appropriately chosen contacts as bonds (chords). We generate contact maps by standard means. Those contact maps include hydrogen bonds, van der Waals interactions, etc. In general proteins have much longer chains than RNA (apart from ribosomes), so it is expected that their genus can attain much larger values, and the genus trace should capture fine details of their structure. One important result of our analysis is that, similarly as in the case of RNA, the genus trace reveals information about the domain structure of proteins. In particular we found, that it predicts cooperative folding pattern in multi-domain proteins.

**Figure 6:**
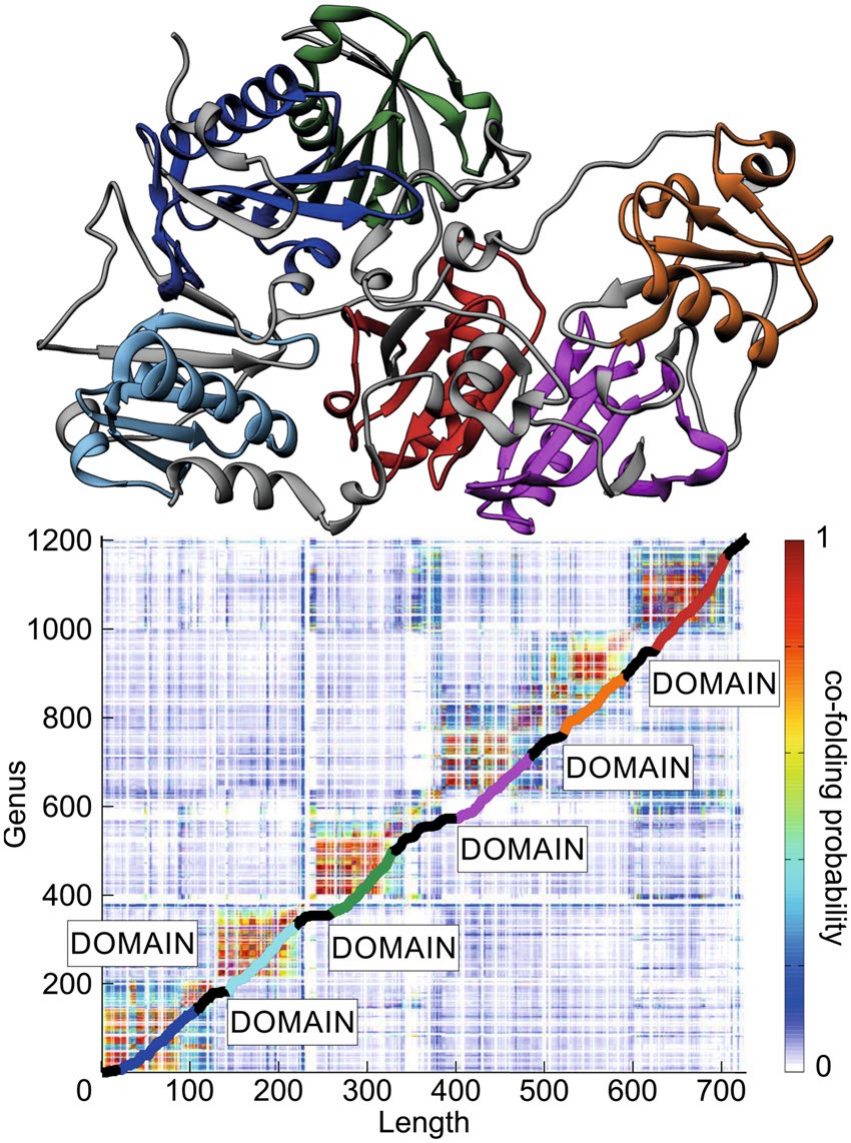
The genus trace detects the domain structure of proteins. We illustrate this statement in the case of gelsolin protein (PDB ID 1d0n), whose cartoon representation is shown in the top panel. In the bottom panel the genus trace for this protein (the main curve) is superimposed on the heat map, which represents the degree of cooperativeness of the folding process, and indicates elements of the protein which are formed at the same time. The degree of cooperativeness is indicated with color bar, and cooperative regions form red-yellow squares. Flat regions (in black) in the genus trace detect unstructured regions between domains, and colored regions indicate domains (shown in the top panel). Left borders of flat regions and domains are well correlated with red-yellow squares in the heat map, which indicates cooperative folding inside domains.

We illustrate the above statements in the example of the gelsolin protein – an actin-binding protein regulating actin filament assembly – of PDB ID 1d0n. This protein comprises six structurally related domains, identified via the Pfam database. In the cartoon in Fig. 6 (top), each of those domains (according to the Pfam classification) is shown in different color: blue, cyan, green, magenta, orange, and red; and linkers between domains are shown in grey. In the bottom panel of Fig. 6, the genus trace for this protein is superimposed on the heat map, which represents cooperative folding pattern.

Note first, that the genus trace plot in Fig. 6 (bottom panel) has clear characteristic features: in regions (denoted in black) separating neighboring domains it flattens, similarly as we noticed earlier for ribosome structures. In result, various parts of the genus plot, shown in the same colors as domains in the top panel, are clearly assigned to these corresponding domains. Therefore the genus trace encodes the domain structure of proteins. Note that the genus of the entire protein chain is very large, of the order of 1200, so it is sufficient to accommodate jumps in its value, and undoubtedly more refined information can be extracted from the genus trace.

Furthermore, we found that the genus trace encodes kinetic information about domain formation. To this end we conducted successful kinetic folding simulations of this protein with a coarse-grained native-centric model^17^. We constructed a method (described in detail in the SI Appendix) to detect areas in the protein which fold at the same time. The degree of cooperativeness of the folding process is shown in the heat map, shown in the background of the genus plot, in the bottom panel in Fig. 6. The formation of each domain is represented by red and yellow dots, which form larger squares in this figure. It is clearly seen, that locations of these squares are correlated with the genus trace; in particular flat, black regions of the genus trace plot correspond to left borders of red-yellow squares in the heat map. Thus the simplest genus analysis predicts cooperative folding – formation of secondary structures to precipitate cooperative folding of a given domain. Such cooperative folding was observed experimentally for smaller partially disordered multi-domain proteins^18^.

As we see, already the simplest genus analysis in proteins reveals nontrivial information about their structure and dynamics. Such an analysis should reveal interesting information also for other models of proteins, ones that take into account other contact maps. For example, a more complicated fat-graph model of proteins was introduced in^9^, where two types of hydrogen bonds were considered. Yet another classification of hydrogen bonds into several distinct classes was introduced in^19^, which is also worth analyzing by means of genus traces. Furthermore, a genus analysis would be of advantage in the analysis of complexes, e.g. chaperons, proteasomes, or capsides built of dozens of proteins.

## Discussion

In this work we introduced the notion of the genus trace, a function that captures the genus assigned to various subchains of a given chain (after representing each such subchain as a chord diagram). The genus trace is a measure of complexity of the structure of bonds connecting various elements of a given chain; for example, in RNA it captures the complexity of pseudoknots. It also gives a way to quantify how much more complicated a biomolecule is than its nested secondary structure alone would indicate. The genus trace also enables one to imagine how an observer, who is sitting at the “synthesis end”, can see new details about how the molecule is folded up during synthesis.

We found that the genus trace captures important information for various types of biomolecules. First, its plot has obvious plateaus, which indicate the domain structure of RNA and proteins. Second, the genus trace captures specific properties of various types of RNA base pairs in the Leontis-Westhof classification: sugar-sugar interactions are seen to significantly increase complexity of RNA chains (as expected, and as follows also from Fig. 3), while Hoogsteen-Hoogsteen interactions are rare (which follows simply because their number in the PDB is low). Third, the overall complexity buildup of the genus trace is not linear, and in many cases the genus raises faster per unit length at the 3′ end than at the 5′ end (as is clearly seen e.g. in Figs 4 and 5); this follows from the geometry of RNA motifs and how they often pack in 3D along the grooves of one another.

Apart from introducing the genus trace, we have also conducted a thorough analysis of the genus of the full RNA chains (significantly improving the analysis in^3^, which took into account only canonical base pairs). We have found that the total genus per unit length is roughly linear. On the other hand, artificial RNA structures have lower genus (and thus lower genus per length) compared to natural structures, which follows because they still lack the structural complexity in 3D that natural structures have.

Furthermore, based on computer simulations we found that the genus trace can be used to predict cooperative folding of small local regions of a protein, which leads to domains formation inside multi-domain proteins. This confirms that the genus trace encodes also some dynamical information about biomolecules.

Finally, let us discuss some other ideas what the genus and genus trace could be useful for. In particular, it should enable the characterization of dynamical properties of biomolecules. As in the first example, note that in riboswitches an interplay between the structural dynamics and the genus should be observed. The structural transitions of a riboswitch will cause the genus to increase or decrease, and this is what allows energy from small structural changes like ligand-binding to be transduced into larger structural rearrangements inducing the formation of a terminator stem. Structures with higher genus might have more energy available in a form that is able to be moved around more easily, and then used for mechanical energy. As the second example, note that for ribozymes in the crystal structure we see the genus only for a single locked state – but in fact they are dynamic structures that shift between multiple states in a ratcheting-like motion. During such a motion many connections that contribute to genus are formed and broken. Analysis of behavior of genus and interactions between two subunits, or between different proteins or RNA molecules, would also characterize their dynamical properties.

The genus trace should become an important tool in analysis and classification of various biomolecules, as well as entire complexes, which would involve more than one backbone, and whose topological structure would be even more interesting. The information encoded in the genus trace would be important also beyond the realm of biomolecules, e.g. for various types of polymers. We are convinced that the genus trace deserves further studies and it should find plenty of applications.

## Materials and Methods

### Protein dynamics

The dynamics of the gelsolin protein (PDB ID 1d0n) was conducted using the Gromacs v4.5.4 with a Gaussian-type contact potential. We used the standard C_*α*_ model proposed by the SMOG server^17^ with the Shadow contact map. The folding was studied through constant temperature molecular dynamics simulations using a Nose–Hoover thermostat with coupling 0.025. Trajectories were run near expected folding temperature T_*f*_. More details about simulations and the method to detect domains is described in the Supplementary Information.

## Electronic supplementary material

Supplementary material
