# Supplementary material for: Genus trace reveals the topological complexity and domain structure of biomolecules
Source: Sci Rep. 2018 Dec 3;8:17537. doi: 10.1038/s41598-018-35557-3 (PMC6277428; doi:10.1038/s41598-018-35557-3)
Supplement: Supplementary file 1 — Supplementary material [file 41598_2018_35557_MOESM1_ESM.pdf]

Genus trace reveals the topological complexity and domain structure of biomolecules

Supplementary Material

Sebastian Zając, Cody Geary, Ebbe Andersen, Pawel Dabrowski-Tumanski,
Joanna I. Sulkowska, Piotr Sułkowski

Molecular dynamics simulations of the gelsolin protein (PDB code 1d0n)

Molecular dynamics simulations of the gelsolin protein have been conducted using the Gromacs v4.5.4 with Gaussian contact potential [1]. The model that we use is the standard C_α model proposed by the SMOG server [2] with the Shadow contact map. The folding of proteins has been studied through constant temperature molecular dynamics simulations using a Nose–Hoover thermostat with coupling 0.025.

Folding of the gelsolin protein (PDB code 1d0n)

Regions of the gelsoin protein which fold at the same time have been detected based on folding simulations, following a method described below.

1. Obtain unfolded conformations of gelsolin. To ensure robustness, several different unfolding conformations were obtained.
2. Perform a sufficient number of folding simulations, starting from the unfolded structures obtained in the previous point. To ensure robustness of results, the simulations should be carried out at a few different temperatures.
3. In each folding simulation identify the points in time, where some tertiary structure is formed. These points divide the folding trajectory to time intervals.
4. Identify which residues form given tertiary structure – this gives a set of “co-folding" residues for each time interval identified in the previous point.
5. Calculate, how often residues “co-fold”, i.e. what is the statistical probability, that two given residues are in the same set distinguished in the previous point.
6. The results are presented in a matrix, where the degree of a cooperativity is visualized via a heat-map.

The details of the analysis are as follows:

Ad. 1. The unfolding of the gelsolin protein (PDB code 1d0n) has been performed in coarse grained (C_α) structure based model using the Gaussian potential. The structure of gelsolin was unfolded in high-temperature simulations, from which 7 non-correlated (separated by at least 2500 frames) structures have been extracted.

Ad. 2 Folding was performed within the same model in four different temperatures $T < T_f$ (where T_f is the equilibrium temperature between folded and unfolded phase). Depending on the temperature 30-70 constant temperature simulations were performed, giving in total 200 folding trajectories. The protein was regarded as folded if the fraction of native contacts formed Q was larger than 0.9, where $Q = 1$ indicates that all native contacts are formed. In such case, the simulation was terminated.

Ad. 3 In order to identify points where the tertiary structures were locally formed, first the fraction of native contacts Q in each frame was calculated. Then, the $Q(t)$ trace was smoothed with running average with a window of 20 frames. Next, the jumps of the smoothed trace $Q_{ave}(T)$ were obtained. The jumps were defined as the points in which the trace $Q_{ave}(T)$ increased by at least 0.05 between consecutive frames. This divided the folding mechanism into time intervals, which were subjected to subsequent analysis (see Fig. 1).

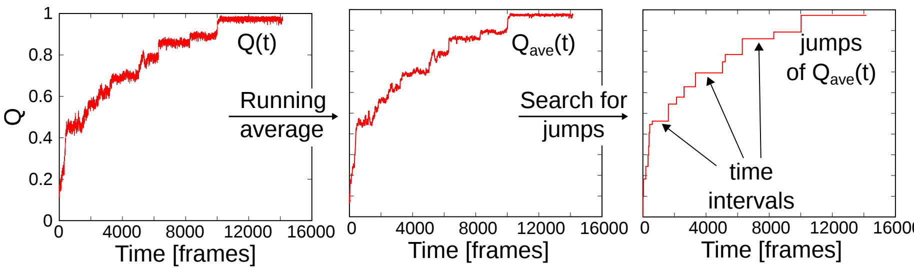

Figure 1: **Illustration of the steps performed to find "points" in which some tertiary structure is "locally" formed.** From left to right: calculation of $Q(t)$, smoothing $Q(t)$ with running average, identification of jumps in the trace $Q_{ave}(t)$. The fragments between the jumps, represented as horizontal intervals in the right-most plot, are the time intervals subjected to further analysis. Three exemplary time intervals are denoted by the arrows.

Ad. 4 For each time interval obtained in the previous point the residues which were "locally folded" were identified. By "locally folded" residue we mean the residue which:

- has at least 4 contacts in the native structure;
- at least 50% of contacts were formed in at least 75% of the frames from analyzed time interval.

After prescription of the set of residues "locally folded" in each time interval, the residues which were "locally folded" between each two intervals were extracted. To this end, we simply compare the sets related to consecutive time intervals. The residues obtained were regarded as “co-folding" in the given simulation. For each simulation, a few sets of “co-folding" residues were obtained (as many as time intervals).

Ad. 5 Next, for each pair of residues we calculated how often a given pair of residues is in the same set of “co-folding" residues. The results were normalized to the number of trajectories (200), as each residue appears in one set only in a given trajectory. This procedure gives a matrix whose entries are the probability that two residues “co-fold”.

Ad. 6 The matrix is presented as a heat-map using Gnuplot 4.6.

References

[1] Lammert H, Schug A, Onuchic JN *Robustness and generalization of structure based-models for protein folding and function*, Proteins: Struct., Funct., Bioinf. (2009) 77(4), 881-891.

[2] Noel JK, Levi M, Raghunathan M, Lammert H, Hayes RL, Onuchic JN, Whitford PC *SMOG 2: A Versatile Software Package for Generating Structure-Based Models*, PLoS Comput Biol (2016) 12(3), e1004794.
